# Mitogen-activated protein kinase 4 is obligatory for late pollen and early fruit development in tomato

**DOI:** 10.1093/hr/uhac048

**Published:** 2022-03-14

**Authors:** Jie Wang, Mengzhuo Li, Shibin Zhuo, Yue Liu, Xiaolin Yu, Sidra Mukhtar, Muhammad Ali, Gang Lu

**Affiliations:** 1 Department of Horticulture, Zhejiang University, Hangzhou 310058, China; 2 Ningbo Academy of Agricultural Sciences, Ningbo 315000, Zhejiang, China; 3 Directorate of Agriculture Research, Agricultural Research Institute Tarnab, Peshawar, Pakistan; 4 Key Laboratory of Horticultural Plant Growth, Development and Quality Improvement, Ministry of Agricultural, Zhejiang University, Hangzhou 310058, China

## Abstract

Mitogen-activated protein kinase (MAPK) cascades are universal signal transduction modules regulating vegetative and reproductive development of plants. However, the molecular mechanisms of the *SlMPK4* gene in tomato pollen and fruit development remain elusive. *SlMPK4* is preferentially and highly expressed in tomato stamens and its mRNA levels increase during early flower development, peaking at the mature pollen stage. Either up- or downregulation of *SlMPK4* expression had no significant effect on tomato vegetative growth. However, RNAi-mediated suppression of *SlMPK4* caused defects in pollen development, resulting in pollen abortion. The aborted pollen grains were either malformed or collapsed and completely lacked viability, resulting in a predominantly reduced fruit set rate in RNAi lines compared with control and overexpressing transgenic plants. Interestingly, seed development was inhibited in RNAi lines. Moreover, >12% of emasculated RNAi flowers developed seedless fruits without pollination. Anthers can produce typical microspore mother cells as well as uninucleate microspores, according to cytological investigations, while binucleate pollen ceased to produce typical mature pollen. Pollen abortion was further confirmed by transmission electron microscopy analysis at the binucleate stage in RNAi plants. The exine layer in aberrant pollen had a normal structure, while the intine layer appeared thicker. Suppression of *SlMPK4* affects the transcript level of genes related to cell wall formation and modification, cell signal transduction, and metabolic and biosynthetic processes. A subset of genes that may be putative substrates of plant MAPKs were also differentially changed in RNAi transgenic flowers. Taken together, these results suggest that *SlMPK4* plays a critical role in regulating pollen development and fruit development in tomato plants.

## Introduction

The generation of male gametophytes in flowering plants is a complicated process, closely controlled spatiotemporally, that includes two phases: microsporogenesis and microgametogenesis [1, 2]. Microsporogenesis is the process in which archesporial cells produce primary sporogenous cells by periclinal division and then develop to form microsporocytes (also known as pollen mother cells). Then, during meiotic division, pollen mother cells produce tetrads of microspores, which are surrounded by a callose wall. Microgametogenesis begins with microspores released from the tetrads; the released microspores undergo unequal mitosis, forming bicellular pollen composed of a large vegetative cell and a small generative cell. The generative cell then undergoes a single round of mitotic cell division to give rise to two sperm cells after anthesis and pollen tube growth, whereas the vegetative cell does not divide again. At anthesis, pollen grains are released from the anther to the stigma for germination and fertilization [3]. In angiosperms, one of the two sperm cells fuses with the egg cell to form the zygote, which develops into an embryo, and the second sperm cell fertilizes the polar nuclei to give rise to the primary endosperm cell. With the development of the embryo and the endosperm, the ovules enlarge into seeds, and the maternal tissues surrounding the embryo sac form the seed coats [4].

Plant development is subjected to a lot of external stimuli and endogenous developmental signals, including pollen and seed developmental signals. Previous research proved that plant signal transduction relies on a large number of protein phosphorylation and dephosphorylation reactions [5]. Genetic and molecular studies have demonstrated a network of genes during pollen and seed development that are components of signal transduction pathways like the mitogen-activated protein kinase (MAPK) pathway [6, 7]. In eukaryotes, the MAPK cascade has arisen as a universal signaling module that connects many receptors/sensors to cellular and nuclear responses [8]. MAPK signaling modules, which usually consist of three consecutively active phosphorylation protein kinases, are evolutionarily conserved in eukaryotes, such as MAPKs, MAPK kinases (MAPKKs), and MAPKK kinases (MAPKKKs) [9]. Recent evidence shows that plant MAPKs are involved in regulating reproductive development. In several plant species, various MAPKs have been reported to be expressed during cell proliferation and differentiation, such as pollen development and maturation [10]. The Ntf4 MAPK has been proven to be regulated during pollen maturation as well as at the beginning of pollen germination [11]. *MPK3* and *MPK6* govern cell proliferation and differentiation during anther formation in *Arabidopsis* plants [12], and also function in ovule development [13]. Furthermore, it has been established that *AtMPK6* is involved in seed formation [14]. According to recent research, *AtMPK4* is involved in various developmental programs, including meiotic cytokinesis during pollen formation [15, 16]. However, there is little evidence available to support the significance of the actual functions of other MAPKs that are actively involved during pollen development.

Tomato is a vital commercial vegetable crop and is a model plant species for investigating reproductive development in plants. However, high temperature, drought, and salt stress have a significant impact on tomato flower and fruit development. These adverse environmental factors usually cause abnormal pollen development and/or pollen abortion, and consequently result in poor pollination, fertilization, and fruit set in tomato. An earlier study in our laboratory identified and characterized the transcript levels of all *Solanum lycopersicum* MAPK putative genes during flower development against abiotic stresses [17]. However, previous studies focused on three tomato MAPK genes (*LeMPK1*, *LeMPK2*, and *LeMPK3*) and their functions in defense response and stress tolerance [18, 19]. A recent study showed that MAPK1/2 can be activated after RBOH1-dependent H_2_O_2_ production and contribute significantly to acclimation-induced cross-tolerance in tomato plants [18]. MAPK2 also takes part in brassinosteroid-induced H_2_O_2_ generation and tolerating stress [20]. Additionally, *SlMAPK7* is crucial in tomato pollen formation, as we discovered in prior work, possibly by regulating tapetum degradation; *SlMPK20* modulates sucrose and auxin metabolism as well as signaling to govern postmeiotic pollen formation in tomato [21, 22].

The involvement of *SIMPK4* in postmeiotic pollen development in tomato is described in the current study. *SlMPK4* was found to be highly expressed in developing anthers. RNAi-mediated downregulation of *SlMPK4* resulted in abnormal pollen grains that were malformed or collapsed and lacked viability, leading to reduced fruit set rate when compared with control plants. Moreover, all these changes were associated with greatly decreased seed production. On the basis of our findings, therefore, we propose that *SlMPK4* plays a critical function in late pollen and fruit development in tomato.

## Results

### Phylogenetic and sequence analysis of *SlMPK4*

The phylogenetic tree was constructed for MAPKs from tomato (*S. lycopersicum*), cucumber (*Cucumis sativus* L.) rice (*Oryza sativa*), and arabidopsis (*Arabidopsis thaliana*) (Supplementary Data Fig. S1). The results demonstrated similar arrangements when tomato or arabidopsis was used alone and members of the similar groups clustered together. The tomato genome encodes 19 MAPK proteins. However, the phylogenetic analysis of SlMAPK protein showed that SlMPK4 is closely associated with SlMAPK7, sharing 75% amino acid sequence similarity, and both fall into subgroup B. A BLAST search against the NCBI database showed that SlMPK4 shares 97% sequence identity with NRK1/Ntf6 of *Nicotiana tabacum* and 77% with AtMAPK13 (Q9LMM5.1), while AtMAPK4 (NP_192046.1) is ~75% similar to the query sequence SlMPK4. The sequences that exhibited >75% similarity were regarded as homologous genes of *SlMPK4.*

### Expression patterns of *SlMPK4*

**Figure 1:**
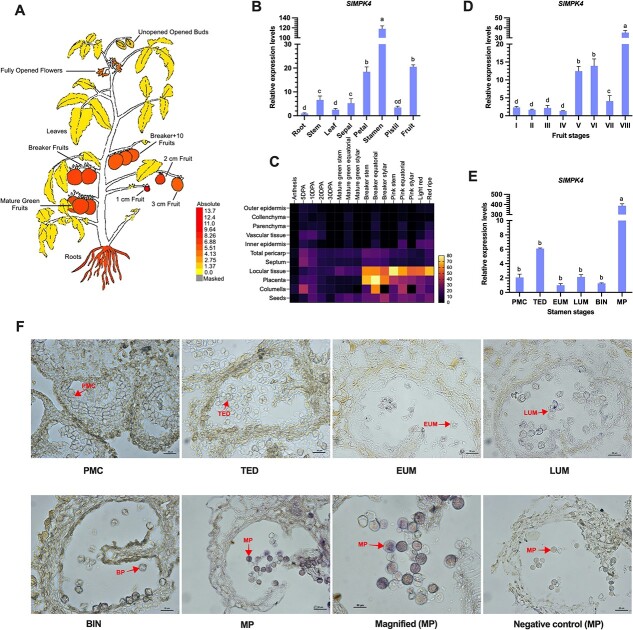
Spatial and temporal expression pattern of *SlMPK4* in tomato. **a** Predicted expression level of *SlMPK4* in different parts of the tomato plant. Results are presented in a heat map; yellow color represents minimum and red color represents maximum expression. **b** qRT–PCR analysis of the expression pattern of *SlMPK4* in different tissues: root, stem leaf, sepal, petal, stamen, pistil (opened flower), and fruit (green mature fruit). **c** Heat map showing the predicted transcript level of *SlMPK4* in different tissues of tomato fruit. The results were retrieved from the tomato database [23]. **d***SlMPK4* mRNAs at different developmental stages of tomato fruit: I (fruit at anthesis), II (fruit diameter 1–2 mm), III (fruit diameter 3–4 mm), IV (fruit diameter 5–6 mm), V (fruit diameter 7–8 mm), VI (green mature fruit), VII (break fruit), and VIII (mature fruit). **e***SlMPK4* expression in stamens at stage PMC (pollen mother cell; 1.8–2.4 mm buds), TED (tetrad; 2.5–3.5 mm), EUM (early uninucleate; 3.6–4.5 mm), LUM (late uninucleate; 4.6–6.5 mm), BIN (binucleate; 6.6–7.5 mm), and MP (mature pollen; flower >7.5 mm). **f** Cross-sections of tomato anthers *in situ* hybridized with *SlMPK4* antisense probe at six developmental stages. Scale bars = 25 μm*.* Plotted values are mean ± standard deviation (*n* = 3 biological replicates), separated using Duncan’s multiple range test (*P* < .05); means with different lower-case letters represent significant differences.

To illuminate the expression characteristics of the *SlMPK4* gene in various vegetative and reproductive tissues of tomato, we systematically carried out *in silico* and qRT–PCR analyses, which exhibited higher variance in the transcript of *SlMPK4* in distinctive tissues and stages in all selected tissues (Fig. 1). *In silico* analysis data were retrieved from a publicly available transcriptomic database of tomato (cv. ‘Heinz’) [23]. The transcript of *SlMPK4* in the tomato plant revealed marked differences among the different tissues, as demonstrated in Fig. 1a, which displays the indexes of expression levels, which ranged from yellow (0) to red (13.7). Moreover, the expression level was highest (13.7) in fruits, closely followed by flowers and roots. To further authenticate the transcript level of *SlMPK4* in different reproductive and vegetative tissues (root, stem, leaf, sepal, petal, stamen, pistil, and fruit) we cultivated cv. ‘Micro-Tom’ in normal conditions. The qRT–PCR analysis showed that highly significant transcription was observed in the stamen (118-fold) relative to other organs (Fig. 1b), suggesting its potentially important role in stamen development. In fruit, the transcript levels of *SlMPK4* were also higher (20.5-fold).

To illuminate the expression of *SlMPK4* in tomato fruit in detail, first a set of microarray data was attained from the tomato database [24] (Fig. 1c), and then qRT–PCR analysis was performed for eight developmental stages (Fig. 1d). As the data presented in the heat map demonstrate, *SlMPK4* was expressed predominantly in the late stages of fruit development, which shows its dynamic role in the development and regulation of tomato fruit. Moreover, the result obtained from qRT–PCR analysis showed that *SlMPK4* was expressed predominantly in mature fruit (stage VIII), with its mRNA level being >35-fold relative to other stages, closely followed by stages VI (>13-fold) and V (>12-fold). However, the microarray and qRT–PCR data exhibited a correlation in most tissues and showed a certain degree of tissue specificity, while the dissimilar transcription might reflect variations in biological materials. Therefore, the overall tissue-specific expression of *SlMPK4* suggests a potential biological function in tomato fruit development.

Furthermore, to deeply authenticate the expression of *SlMPK4* in tomato stamen, six developmental stages were studied, i.e. pollen mother cell, tetrad, early uninucleate microspore, late uninucleate microspore, binucleate pollen, and mature pollen. The results revealed that a remarkable expression level (387-fold) was noted in the mature pollen stage, which shows its vital function at the mature pollen stage of development (Fig. 1e). In order to complement the qRT–PCR analysis and to elucidate the spatial transcription of *SlMPK4*, we systematically approached *in situ* hybridization using flower bud sections at different stages as defined above (Fig. 1f). The findings revealed that *SlMPK4* expression was strongly detected in the mature pollen grains as compared with sense control of mature pollen (Fig. 1f), while the mRNA was undetectable in early anthers, including pollen mother cell, tetrad, early uninucleate microspore, and binucleate stages. The integrated investigation indicated that the expression of the target gene *SlMPK4* plays an important function in late stamen development in tomato.

**Figure 2:**
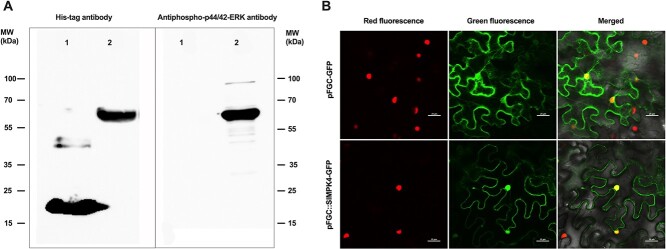
SlMPK4 protein analysis and confirmation. **a** Western blot of purified SlMPK4 fusion protein using His-tag antibody. 1, Purified protein induced in the empty pET32a (+) vector, which contains a Trx-Tag, small His-Tag, and S-Tag. The total molecular weight of the purified protein is ~20.5 kDa. 2, Purified SlMPK4 protein fused with a Trx-Tag, small His-Tag, and S-Tag. **b** Subcellular localization assay of SlMPK4. Expression of SlMPK4 fused with red and GFP via *Agrobacterium*-medicated transient expression in *N. benthamiana* epidermal cells. The positions of nuclei are shown by the H2B-RFP marker. Scale bars = 25 μm.

### SlMPK4 coding protein and its subcellular localization

As shown in Supplementary Data Fig. S1, the amino acid residues of tomato SlMPK4 and its homologs in various crops were highly conserved, whereas the 11 different domains (I–XI) of MPK4 were positioned in a highly conserved region of 300 amino acid residues at the N-terminal portion. Moreover, domain VIII, which encodes threonine and tyrosine, named the TXY motif, is a phosphorylated activation of SlMAPK proteins. Notably, the SlMPK4 protein sequence carries four types of special subdomains: substrate-binding site, active site, ATP binding site, and activation loop (A-loop). Western blot analysis using anti-His antibody showed that the protein induced in the empty pET32a (+) vector was ~20 kDa and the purified SlMPK4 fusion protein was ~64 kDa, indicating that the molecular mass of the SlMPK4 protein characterized by SDS–PAGE was 44 kDa (Fig. 2a). The mass was similar to the one predicted by the deduced amino acid sequence in the online tool ExPASy (http://web.expasy.org/compute_pi/), which is 43.2 kDa. Furthermore, to verify whether the tomato SlMPK4 has MAPK activity, the kinase activity assay was detected using anti-pTEpY as previously described [25, 26]. Protein bands were detected in the presence of an enhanced chemiluminescence reagent, which confirmed that SlMPK4 encodes a protein that has MAPK activity. Additionally, to authenticate the protein localization of SlMPK4 *in vivo*, we transiently transformed the pFGC::SlMPK4-GFP (GFP, green fluorescent protein) fused plasmid into *Nicotiana benthamiana* leaves. Confocal laser micrographs showed that GFP was observed in the nucleus, cytoplasm, and cell membrane (Fig. 2b).

### Generation of *SlMPK4*-overexpressing and RNAi transgenic tomato

The function of *SlMPK4* was investigated by RNAi-mediated gene silencing and overexpression in tomato variety ‘Micro-Tom’. Two RNAi constructs were prepared using a 400-bp fragment of the *SlMPK4* cDNA sequence (Supplementary Data Table S1), driven by a constitutive CaMV 35S promoter and the tomato pollen-specific promoter *LAT52* [27]. Multiple transgenic lines were generated and confirmed through PCR detection of genomic DNA and β-glucuronidase (GUS) histochemical assay. The obtained positive transgenic lines included p35S::*SlMPK4*-RNAi (35S:M4i lines), pLAT52::*SlMPK4*-RNAi (LAT:M4i lines) with nine lines each, and six p35S::*SlMPK4* (OE:M4 lines) overexpressing lines (Supplementary Data Fig. S2).

The qRT–PCR results showed that *SlMPK4* transcription was decreased in the stamens of p35S:4i and pLAT:4i lines as compared with control plants (Supplementary Data Fig. S2A and B), although the extent of inhibition differed among various independent transgenic lines. By contrast, *SlMPK4* transcription was substantially increased in OE:M4 lines (Supplementary Data Fig. S2C). Furthermore, we measured the transcript levels of *SlMPK4* in different tissues of RNAi plants to investigate the variation. The 35S:M4i-15 line displayed a significant reduction in *SlMPK4* mRNA levels in all tissues except the root, while a marked decrease was observed in the stamen (Supplementary Data Fig. S2D). However, the transcript level of *SlMPK4* in LAT:M4i-2 stamen was markedly lowered relative to other tissues and also to 35S:M4i-15 stamen (Supplementary Data Fig. S2D and E).

As presented in Supplementary Data Fig. S1A, the amino acid sequence of SlMAPK7 had 75% similarity with SlMPK4; however, to eliminate the co-expression risk we detected the transcript levels of *SlMAPK7* in RNAi lines. The qRT–PCR analysis confirmed that the transcript of *SlMAPK7* was not altered in *SlMPK4* knockdown tomato plants as compared with control plants (Supplementary Data Fig. S2F). Based on knockdown efficiency and overexpression, we selected two independent representative lines from each of p35S::*SlMPK4*-RNAi (35S:M4i-15 and 35S:M4i-20), pLAT52::*SlMPK4*-RNAi (LAT:M4i-2 and LAT:M4i-14), and p35S::*SlMPK4* (OE:M4-1 and OE:M4-10) for further study. Moreover, we confirmed the knockdown efficiency of *SlMPK4* through GUS staining by using the above constructs, and the assay revealed strong signals in all tested organs, as visually shown by X-Gluc staining (Supplementary Data Fig. S2G).

### Impact of *SlMPK4* knockdown on tomato pollen development

We next investigated whether *SlMPK4* regulates vegetative and reproductive developments in tomato. Compared with control lines, all transgenic plants exhibited similar vegetative growth except that of 35S-RNAi lines, which were a little shorter than the control plants (Supplementary Data Fig. S3). Meanwhile, the floral organs, including sepals, petals, stamens, and ovaries in OE and LAT-RNAi lines were generally normal compared with the control plants, whereas in 35S-RNAi lines the size of the flower including sepal, petal, and ovary was greater and the style was thicker than in control plants. Interestingly, obvious dehiscence at the top of stamens was observed in 35S-RNAi lines, in contrast to the control (Supplementary Data Fig. S4).

**Figure 3:**
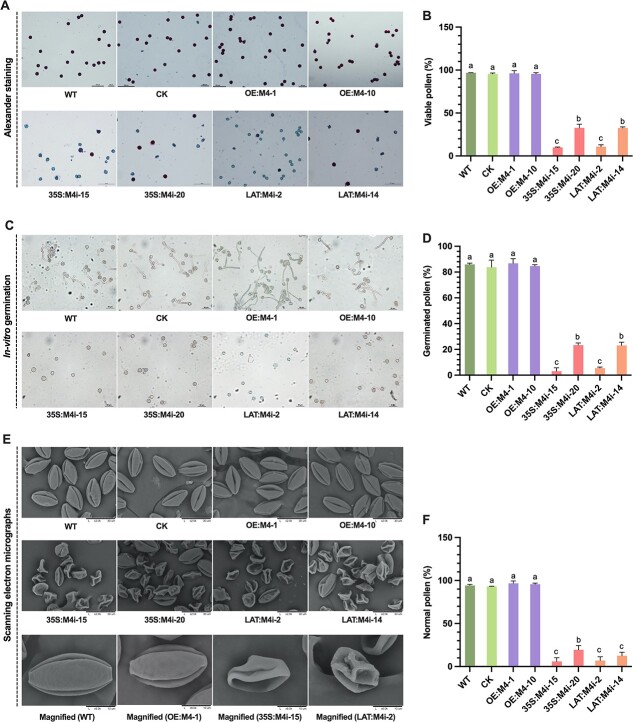
Characterization of mature pollen in transgenic tomato. **a** Alexander staining of mature pollen in WT, control, and transgenic plants. **b** Percentage of viable pollen. **c***In vitro* germination of mature pollen. **d** Germination rate of mature pollen. **e** Scanning electron micrographs of mature pollen. **f** Percentage of normal pollen. Plotted values are mean ± standard deviation (*n* = three biological replicates), separated using Duncan’s multiple range test (*P* < .05); means with different lower-case letters represent significant differences.

The anthers collected from both RNAi (35S:M4i and LAT:M4i) plants at the mature pollen stage (immediately before anthesis) were mostly either empty or contained non-viable pollen. Microscopic examination revealed that the majority of mature pollen grains were shrunk and defective (Fig. 3), while Alexander staining of mature pollen grains implied that the pollen grains in the RNAi lines were mostly non-viable (Fig. 3a). Up to 78–90% of pollen grains from RNAi lines were non-viable, which was far higher than that of control plants (~5%) and OE lines (4%) (Fig. 3b). Consistently, the *in vitro* germination test showed that 95–97% of pollen grains from p35S::*SlMPK4*-RNAi (35S:M4i-15 and 35S:M4i-20) and pLAT52::*SlMPK4*-RNAi (LAT:M4i-2 and LAT:M4i-14) transgenic lines failed to germinate, whereas the germination frequency of p35S::*SlMPK4* (OE:M4-1 and OE:M4-10) and control pollen was >80% under similar culture conditions (Fig. 3c and d).

Scanning electron micrographs revealed that control plant pollen grains were normal, oval-shaped, and with germination apertures distributed evenly (Fig. 3e). However, in anthers of *SlMPK4* RNAi plants up to 96% of the pollen was smaller, malformed, and collapsed (Fig. 3f). The aborted pollen grains, which were markedly reduced in size and deformed, appeared noticeably different from normal pollen from anthers in the control plants. Unlike the *SlMPK4* RNAi plants, significant differences were not observed in the pollen grains from the overexpressing lines and control plants. Only 2% of the pollen grains in the anthers of the overexpressing lines were abnormal. Collectively, pollen germination capacity, vitality, and appearance in the overexpressing lines were typical and consequently similar to those in the control plants. However, downregulation of *SlMPK4* expression in the *SlMPK4* RNAi tomato plants led to an altered pollen developmental phenotype.

### Suppression of *SlMPK4* causes reduction in fruit set and seed number

**Figure 4:**
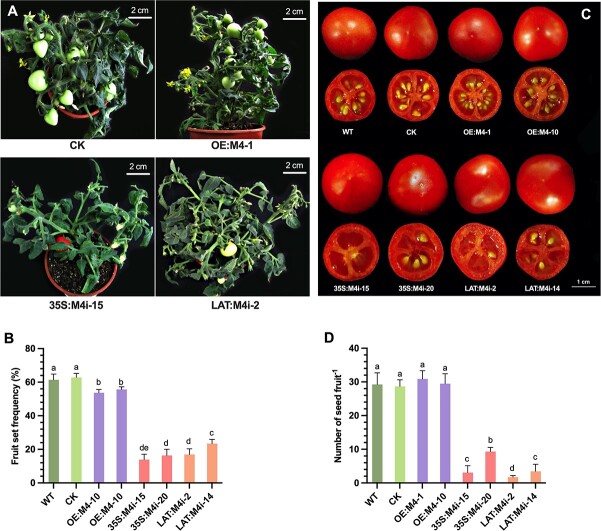
Effect of *SlMPK4* knockdown and overexpression on tomato fruit set and seed number **a** Representative phenotypes of control, *SlMPK4*-knockdown (35S:M4i-15 and LAT:M4i-2) and *SlMPK4*-overexpressing plants. **b** Fruit set frequency. **c** Morphology of mature fruit. **d** Seed number per fruit. Plotted values are mean ± standard deviation (*n* = 3 biological replicates), separated using Duncan’s multiple range test (*P* < .05); means with different lower-case letters represent significant differences.

*SlMPK4* overexpressing and RNAi transgenic plants grew normally and blossomed under controlled conditions. However, the number of fruits per plant significantly declined in RNAi transgenic plants compared with wild-type (WT) plants and OE lines (Fig. 4a and b). There was no discernible change in fruit morphology, including fruit transverse and vertical sections, as well as single fruit weight in OE and LAT-RNAi transgenic plants. Interestingly, examination of the seeds in tomato fruits indicated that the seed number was significantly decreased in 35S:M4i and LAT:M4i fruits (Fig. 4C), which had no more than 10 seeds/fruit or were even seedless. However, WT and control fruit contained >30 seeds/fruit. By contrast, tomato fruits from *SlMPK4*-overexpressing lines developed similar seed numbers to those of control fruits (Fig. 4d). The seed could normally be germinated, indicating that seed development was nearly undisturbed.

**Figure 5:**
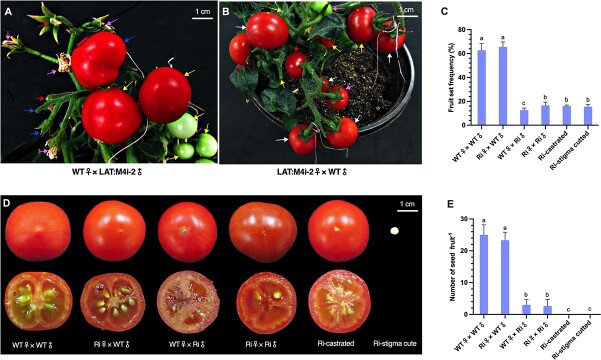
Self- and cross-pollination and emasculation of WT and RNAi plants. **a** Pollination of WT flowers with LAT:M4i-2 pollen (blue arrows), self-pollination (yellow arrows), emasculation (red arrows), and excised stigma (pink arrows) of WT flowers. **b** Pollination of LAT:M4i-2 flowers with WT pollen (white arrows), self-pollination (yellow arrows), and emasculation (red arrows), and excised stigma (pink arrows) of LAT:M4i-2 flowers. **c** Fruit set percentage. **d** Fruit morphology of WT and LAT:M4i-2 plants after self-pollination or emasculation, and reciprocal crosses between them. **e** Number of seeds per fruit. Plotted values are mean ± standard deviation (*n* = 3 biological replicates), separated using Duncan’s multiple range test (*P* < .05); means with different lower-case letters represent significant differences.

To test the fertility of the RNAi plants, crossing tests were performed by pollinating the stigma of flowers on RNAi plants with normal pollen grains of flowers from control plants and vice versa after emasculation the day before anthesis. The RNAi plants were able to bear fruits with normal seeds when pollinated with enough pollen grains from control plants. However, control plants produced only a small number of fruits after flowers were emasculated and pollinated with pollen grains from *SlMPK4* RNAi lines (Fig. 5). Further, to examine the linkage between male sterility and parthenocarpy in RNAi plants, we removed the stamens of RNAi lines and WT plants. The results showed that ~12.3% of emasculated flowers could generate seedless fruits without pollination, while the WT plants were not able to produce any fruits after emasculation, which was similar to our previous results. The above findings indicate that knockdown of *SlMPK4* can lead to a certain degree of male sterility and the generation of partially parthenocarpic fruits.

### Pollen abortion occurred during late stage of anther development in *SlMPK4* RNAi plants

**Figure 6:**
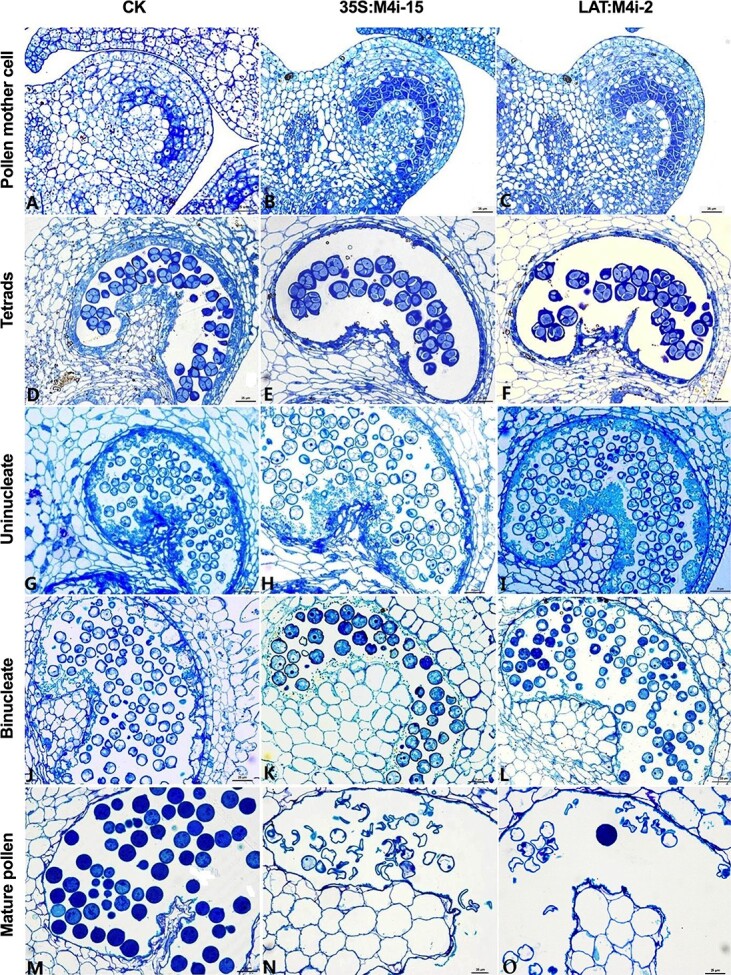
Transverse sections of anthers at five stages (pollen mother cells, tetrads, uninucleate, binucleate, and mature pollen) from control, 35S:M4i-15, and LAT:M4i-2 plants. Scale bar = 25 μM.

It was revealed that pollen development seemed to be normal initially in the RNAi plants. In both WT and RNAi (35S:M4i-15 and LAT:M4i-2) plants, typical pollen mother cells were visualized within the anther locules, entered meiosis, and produced classical tetrads (Fig. 6a–f). The callose wall which bound the haploid tetrads of microspores was enzymatically degraded regularly to release uninucleate microspores from the tetrads (Fig. 6g–i). Individual uninucleate microspores developed to binucleate pollen after pollen mitosis-1 (Fig. 6j–l). From binucleate to mature pollen stage, whereas the binucleate pollen developed into normal mature pollen grains in the control plants, the binucleate pollen in RNAi plants failed to form normal mature pollen grains; instead some empty and anomalous cells were observed in the locule (Fig. 6m–o). Moreover, the phenotypic differences in mature pollen grains in anther locules were totally in accordance with the differences in transcript levels between the RNAi lines (Supplementary Data Fig. S4).

**Figure 7:**
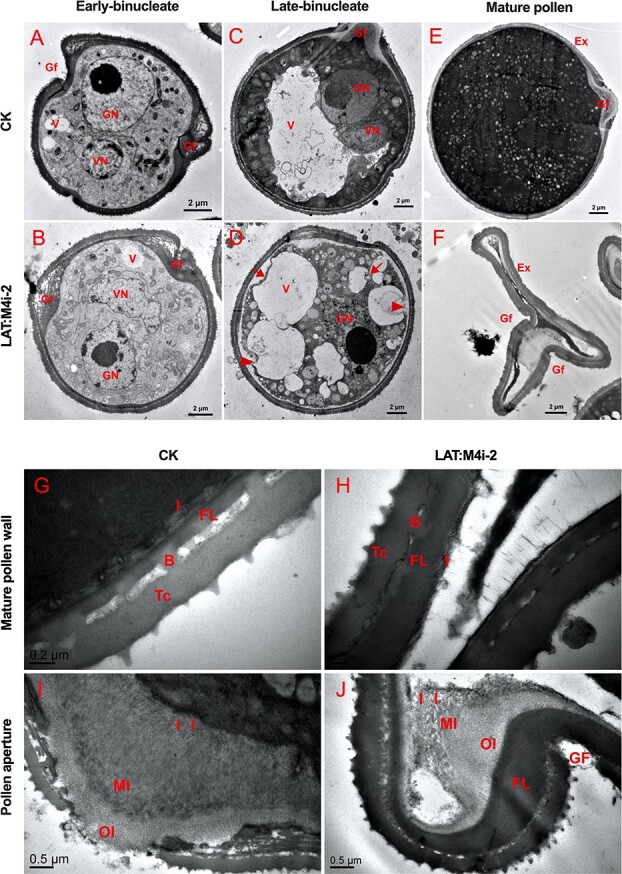
TEM of pollen during late stages of development. **a**, **b** Early binucleate stage. **c**, **d** Late binucleate stage. **e**, **f** Mature pollen from control (**a**, **c**, **e**) and LAT:M4i-2 line (**b**, **d**, **f**). **g**–**j** Magnified images of pollen wall from control and LAT:M4i-2 pollen. Red arrows indicate degrading cytoplasm and arrowheads point to breaks in the plasma membrane. Ex, exine; GN, generative nucleus; VN, vegetative nucleus; V, vacuole; Gf, germinal furrow; I, intine; II, inner intine; MI, middle intine; OI, outer intine; FL, foot layer; B, baculum; Tc, tectum.

In the RNAi anthers, aberrant mature pollen was found (Fig. 6), which prompted us to examine the ultrastructural changes in the microspores using transmission electron microscopy (TEM). TEM showed that highly isochronous and well-developed pollen from control and LAT:M4i-2 anthers contained a well-defined structure at the early binucleate stage (Fig. 7a and b). Besides the cell nuclei, cytoplasm, cytomembrane, cell organelles, and cytoskeleton were formed normally in the control plants. However, the distinct ultrastructural differences between the pollen of control and LAT:M4i-2 plants at the late binucleate and anthesis (mature pollen) stages were observed (Fig. 7c–f). At the binucleate stage in the RNAi anthers, the vacuole fragmented into several small vacuoles, while at mature stage the cellular contents, such as nucleus and cytoplasm, gradually degraded and as result the pollen grains were shrunk (Fig. 7d and f). In LAT:M4i-2 anthers, pollen grains at the mature stage showed a strikingly abnormal phenotype. Moreover, the cytoplasm had disappeared or been smashed due to collapse of the pollen wall, leaving only debris of cellular components in the collapsed pollen cells. In addition, no intact membrane systems were recognized. Finally, the mature pollen displayed degradation of almost the entire cell except for the pollen walls (Fig. 7e and f). Despite the cell walls being preserved, they were wrinkled. Moreover, the shrinking of mature pollen was due to abnormal indentation and folding of the cell wall at the germinal furrow (Fig. 7g–j).

**Figure 8:**
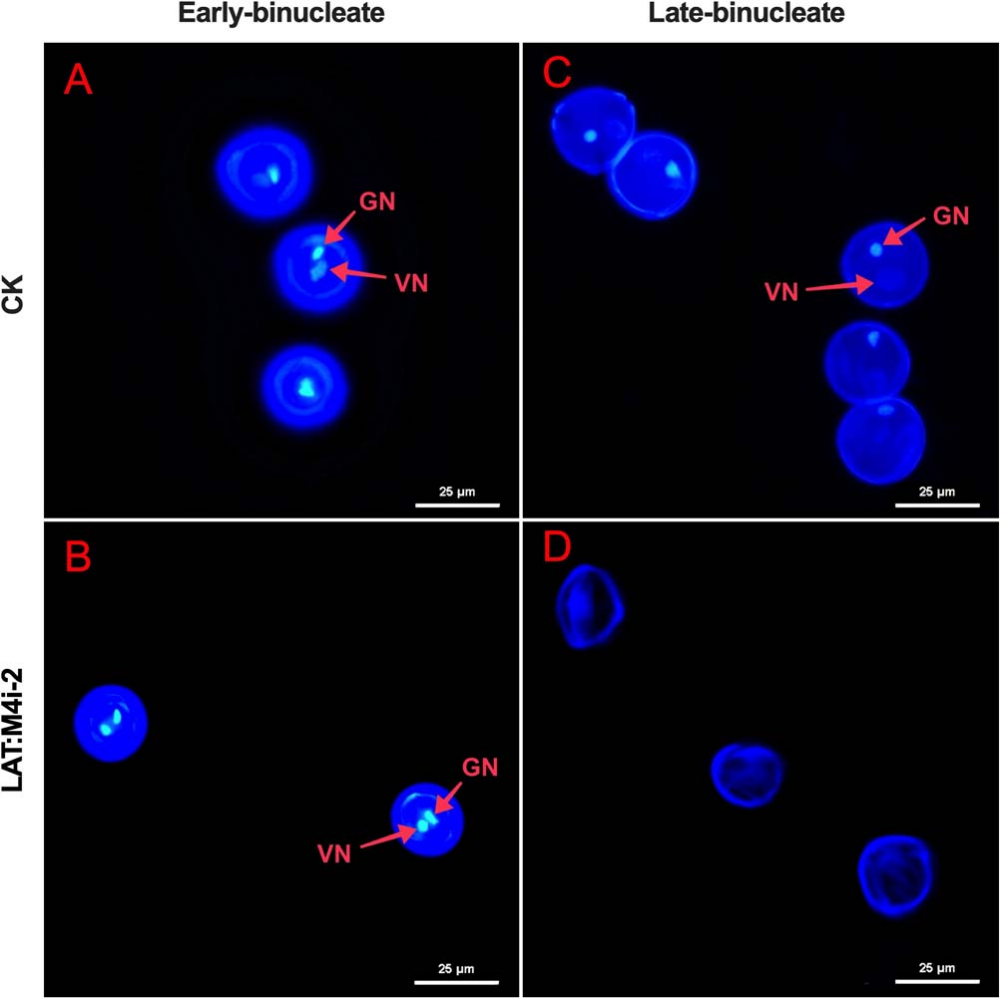
DAPI staining of nuclei of pollen from control and LAT:M4i-2 at binucleate stage. VN, vegetative nuclei; GN, generative nuclei. Scale bar = 25 μM.

Staining with 4′,6-diamidino-2-phenylindole (DAPI) revealed that the binucleate pollen nuclei, including the vegetative nucleus and generative nucleus, were clearly visible in control and LAT:4Mi-2 at the early binucleate stage (Fig. 8a and b), while the two nuclei were not observed in sterile pollen grains of LAT:4Mi-2 lines at the late binucleate stage (Fig. 8c–f), which was in contrast to that of control anthers, which displayed two nuclei at this stage. Taken together, these findings demonstrated that repression of *SlMPK4* expression results in abnormal pollen morphology and biological activity *in vitro*. This indicates that the abortive pollen phenotype that occurred in the transgenic RNAi populations might correlate with the downregulation of *SlMPK4*.

### Identification of *SlMPK4* downstream genes using RNA sequencing analysis

**Figure 9:**
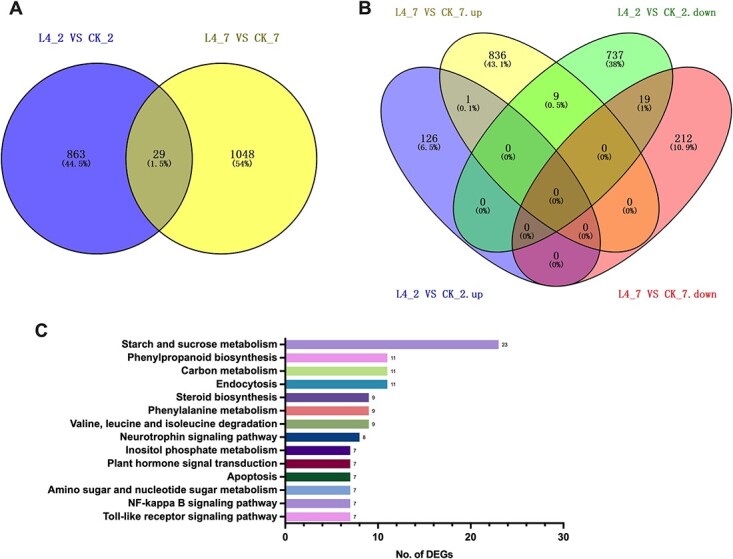
Expression profiling of DEGs in the control (CK) versus RNAi tomato stamens. **a** Venn diagram of DEGs in uninucleate (L4_2) and mature stage (L4_7) stamens. **b** Venn diagram of DEGs up- and downregulated in uninucleate and mature stage stamens. **c** KEGG classification of assembled DEGs at mature stage. Two biological replicates were used in the RNA sequencing experiment.

To further characterize the effect of *SlMPK4* on tomato pollen, RNA sequencing was employed to analyze the expression of genes that were differentially regulated in the LAT-4i RNAi line when compared with control flowers during different floral developmental stages (uninucleate and mature stage) with two biological replicates. Compared with control, totals of 892 and 1077 differentially expressed genes (DEGs) (*P* < .01) were identified in the uninucleate (L4_2) and mature (L4_7) stages, respectively (Fig. 9a). However, only 29 genes were differentially expressed simultaneously at two stages. Whereas in the uninucleate stage a total of 127 upregulated and 765 downregulated genes were found, at the mature stage 846 and 231 up- and downregulated DEGs were found, respectively (Fig. 9b). Among them, nine genes were initially downregulated in the uninucleate stage and then upregulated in the mature stage. Moreover, qRT–PCR analysis of some downstream genes showed a pattern similar to that of the RNA sequencing results (Supplementary Data Fig. S6).

Since pollen abortion occurred at the mature stage, opened flowers were targeted to perform gene ontology (GO) categorization and KEGG classification (Fig. 9). The 846 upregulated genes were mainly divided into 25 subclasses in the molecular function category, among which ‘protein binding’, ‘binding’, and ‘catalytic activity’ were the most enriched with 295, 216, and 209 DEGs, respectively. In the biological process category, the upregulated genes were mainly divided into 47 subclasses, among which the most enriched terms were ‘cellular process’, ‘response to stress’, ‘transport’, and ‘cellular component organization’. In the category of cell component, the upregulated genes were mainly divided into 27 subclasses, among which the most enriched terms were ‘membrane’, ‘plasma membrane’, ‘cytoplasm’, and ‘nucleus’, with 419, 368, 288, and 230 genes, respectively (Supplementary Data Fig. S5A). However, the total number of 996 downregulated genes in the three processes was similar to the number of upregulated genes (Supplementary Data Fig. S5B).

Pathway enrichment analyses were conducted for 1077 DEGs at late stamen development stage, and DEGs were mainly concentrated in 107 metabolic pathways. Statistical analysis was conducted on the 15 metabolic pathways with the highest number of DEGs (Fig. 9c). Among them, the numbers of DEGs significantly enriched in ‘starch and sucrose metabolism’, ‘phenylpropanoid biosynthesis’, ‘carbon metabolism’, and ‘endocytosis’ were the largest. The DEG enrichment of carbohydrate metabolism and cell apoptosis metabolism was statistically analyzed, and the results are shown in Table 1.

In *SlMPK4*-silenced plants, there was a significant change in the transcript levels of a group of genes involved in plant hormone signal transduction, phenylpropanoid biosynthesis, and sucrose metabolism (Table 1). Here, on the one hand, genes participating in different pathways were markedly upregulated, while on the other hand genes contributing to starch and sugar metabolism and hormone signal transduction etc. were significantly downregulated in the LAT:4Mi-2 line when compared with WT. In a recent observation, we found that some genes that controlled pectinesterase were upregulated by a subsequent breakdown and thereby functioning as ubiquitous cell-wall-associated enzymes that facilitate plant cell wall modification. However, with the knockdown of *SlMPK4*, several genes responsible for synthesis and degradation of sucrose were significantly altered. Among them, genes encoding UDP-glucose, play a crucial role in biosynthetic reactions and metabolic pathways, were downregulated. Several different metabolic pathways and biosynthetic reactions include the biosynthesis of polysaccharides such as glycosphingolipids, lipopolysaccharides, starch, and glycogen (Table 1). Aside from the significant impact on other pathways, knockdown of *SlMPK4* substantially reduced the expression level of genes regulating plant hormone signal transduction. These include two genes (Solyc02g092820 and Solyc08g082630) encoding auxin response factor 9 and indole-3-acetic acid-amido synthetase GH3.8, which prevent accumulation of free indole-3-acetic acid (IAA).

## Discussion

### *SlMPK4* plays a crucial role in microgametogenesis

Despite substantial progress towards understanding the important functions of tomato SlMAPKs, we presented a novel and critical role of the *SlMPK4* gene in pollen development in tomato. First, we found that the expression of *SlMPK4* could be found in all selected organs, while the gene displayed a significantly higher transcription in stamens relative to other parts and tissues (Fig. 1b), consistent with previous results [17]. Moreover, *SlMPK4* transcript levels increased during pollen development and reached a peak at the mature pollen stage (Fig. 1). This indicated that it might have a relatively important function during the late development of stamens. In addition, *in situ* hybridization revealed *SlMPK4* mRNA at binucleate and mature pollen stages, whereas it was not observed at the initial stages, in line with a previous study [21]. Second, the knockdown of *SlMPK4* caused aberrant and inviable pollen. During the uninucleate stage, the transgenic plant pollen was normal, but as it matured it became substantially aberrant. Subcellular abnormalities were first observed at the binucleate stage (Figs 6 and 7), while TEM disclosed enormous vacuolation during the early and late binucleate stage. Self-fertilization and reciprocal crosses proved that the pollen phenotype was caused by paternal, not maternal, abnormalities (Fig. 5). These findings, together with *SlMPK4* being expressed in pollen at the binucleate to mature pollen stage (Fig. 1), demonstrate the critical role of *SlMPK4* at the binucleate to mature pollen transition.

**Table 1:** DEGs in tomato stamens of LAT-4i relative to control plants. Log_2_FC, log_2_ fold change.

**Pathway**	**Gene ID**	**Annotation**	**Log**_**2**_ **FC**
**Starch and sucrose metabolism**	Solyc01g066420	Pectinesterase	10.14
	Solyc05g052120	Pectinesterase	10.42
	Solyc05g054360	Pectinesterase	10.10
	Solyc05g052110	Pectinesterase	11.54
	Solyc12g099410	Pectinesterase	10.17
	Solyc06g084620	Pectinesterase	6.60
	Solyc01g099940	Pectinesterase	11.43
	Solyc07g017560	Pectinesterase	11.88
	Solyc01g066360	Pectate lyase family protein	10.18
	Solyc01g066070	Polygalacturonase	7.78
	Solyc06g009200	Polygalacturonase	9.87
	Solyc06g066120	Endoglucanase	7.55
	Solyc12g010200	Glycosyltransferase	3.70
	Solyc04g015530	Polygalacturonase A	3.44
	Solyc10g017620	5-Dehydro-2-deoxygluconokinase 1	5.40
	Solyc09g010090	β-Fructofuranosidase insoluble isoenzyme 2	1.75
	Solyc07g042550	Sucrose synthase	1.72
	Solyc10g085650	β-Fructofuranosidase insoluble isoenzyme 2	−5.06
	Solyc11g071650	β-d-Glucosidase	−6.64
	Solyc03g083550	UDP-d-glucuronate 4-epimerase 2	−2.84
	Solyc07g064180	Pectinesterase	−2.84
	Solyc07g064170	Pectinesterase	−2.47
	Solyc03g115380	UDP-glucose dehydrogenase	−2.94
**Phenylpropanoid biosynthesis**	Solyc11g010960	Alcohol dehydrogenase	−2.12
	Solyc01g058520	Peroxidase 40	−3.18
	Solyc04g080760	Peroxidase	−2.32
	Solyc11g071650	β-d-Glucosidase	−6.64
	Solyc02g084570	Cytochrome P450	2.71
	Solyc03g044100	Peroxidase 5	3.57
	Solyc08g007150	Peroxidase 65	10.25
	Solyc05g006230	Peroxidase	8.93
	Solyc10g050160	Caffeoyl-CoA 3-*O*-methyltransferase	1.61
	Solyc10g086180	Phenylalanine ammonia-lyase	1.85
**Carbon metabolism**	Solyc04g082170	Alcohol dehydrogenase 1	4.05
	Solyc10g083760	Threonine dehydratase biosynthetic	3.64
	Solyc01g018020	Transketolase-like protein	6.06
	Solyc07g044730	3-Hydroxyisobutyryl-CoA hydrolase	8.93
	Solyc07g043680	Enoyl-CoA-hydratase	8.93
	Solyc05g017760	Acetyl-CoA C-acetyltransferase	−2.25
	Solyc10g083540	Threonine dehydratase biosynthetic	−2.74
	Solyc04g082630	Glyceraldehyde-3-phosphate dehydrogenase B	−2.56
	Solyc02g062340	Fructose-bisphosphate aldolase	−3.32
**Plant hormone signal transduction**	Solyc02g092820	Indole-3-acetic acid-amido synthetase GH3.8	−4.06
	Solyc08g082630	Auxin response factor	−3.18
	Solyc06g074320	BZIP transcription factor	−2.56
	Solyc12g009280	Auxin-induced protein-like	9.09
	Solyc05g025920	Auxin-induced SAUR-like protein	2.24
	Solyc01g096810	Ethylene insensitive 3 class transcription factor	1.47
	Solyc07g040990	Protein phosphatase 2C	2.58

### *SlMPK4* is involved in pollen development

There are limited reports on the functions of plant MAPKs in anther/pollen development and male fertility compared with the amount of research regarding the roles MAPKs play during stress responses [28]. A previous study in arabidopsis revealed that *AtMPK3* and *AtMPK6* play crucial roles in cell differentiation during anther development [12]. Recent research showed that the MPK3/MPK6-WRKY34 signaling module functions importantly at the early stage of pollen development [10]. As a homolog of *SlMPK4*, arabidopsis MPK4 is reported to be required for male-specific meiotic cytokinesis. So, the anthers of arabidopsis *mpk4* mutant plants can produce normal microspore mother cells and peripheral supporting tissues; however, the male gametophytes fail to complete meiotic cytokinesis because microspore mother cells cannot generate normal intersporal callose walls after male meiosis [15].

In the current study, we first illuminated the function of an MAPK-*SlMPK4* during pollen development in tomato. Our data showed that there is no apparent change in phenotype either in vegetative or reproductive growth in tomato plants overexpressing *SlMPK4*. Nevertheless, knockdown of *SlMPK4* under the cauliflower mosaic virus (CaMV) 35S promoter and a strong pollen-specific LAT52 promoter during late pollen development [29, 30] resulted in defective pollen. In *SlMPK4* RNAi lines, the pollen grains were malformed and lost biological function. The ultrastructure of the pollen cells from *SlMPK4* RNAi flower buds at different developmental stages indicated that pollen mother cells could proceed with meiosis and microspores with pollen mitosis 1 in an orderly manner, and thus develop normal microspore mother cells, tetrads, uninucleate microspores, and binucleate pollen. However, they failed to form normal mature pollen cells at anthesis and most of their contents rapidly degenerated after the binucleate stage. In some severely abnormal mature pollen cells, only the cell wall persisted, surrounded by empty and anomalous cells. Furthermore, the preserved cell wall of pollen was abnormal as well. Both the exine and intine were aberrantly wrinkled and at the aperture region the intine displayed a thicker wall layer than controls, whereas the exine displayed breakage at some points. In addition, we found that the more severely *SlMPK4* was depressed, the higher the rate of abnormal pollen grains. Therefore, we had plenty of evidence to prove that downregulation of *SlMPK4* results in mature pollen defects. Moreover, we found functions of *SlMPK4* different from those of its homolog in *A. thaliana* during the development of male gametophytes [15]. All of the above data corroborated our prior scanning electron microscopy (SEM) and DAPI staining findings. In general, these data showed that the pollen abortion and ultrastructural abnormalities in *SlMPK4* RNAi lines occurred at the late binucleate stage. Furthermore, the stage at which pollen defects occurred in RNAi lines was consistent with *SlMPK4* expression, which also significantly increased at the later stage.

The CaMV 35S promoter’s activity in anthers and pollen has always been a point of contention. It has been reported that great variability in CaMV 35S promoter activity is detected in relation to anthers and pollen of arabidopsis and tobacco [31]. So, we constructed another RNAi line under a strong pollen-specific promoter, LAT52, and its activity was first observed at the late microspore stage and attained a higher level at the mature pollen stage [32]. However, the CaMV 35S promoter has already been used in *Brassica napus* to construct an antisense RNA vector, and this resulted in insubstantial gene inhibition in flowers [33]. It is noteworthy that LAT52 is thought to be a promoter with the strongest activity in mature pollen [34]. Moreover, all *SlMPK4* RNAi plants driven by the CaMV 35S promoter have nearly the same pollen phenotypes as those driven by the LAT52 promoter. Furthermore, expression profile analysis revealed that the transcript of *SlMPK4* increased by dozens-fold in the flowers and hundreds-fold in the stamens at anthesis while it had a relatively much lower expression at other, earlier developmental stages in both entire flowers and stamens. Hence, it is feasible to use the CaMV 35S promoter in the investigation of pollen or anther genes. In addition, we found that the more severely *SlMPK4* was depressed, the higher the rate of abnormal pollen grains. Therefore, we have plenty of evidence to prove that downregulation of *SlMPK4* results in mature pollen defects.

### Inhibition of *SlMPK4* results in seedless fruits in tomato

Parthenocarpy exists in many species and has been widely studied in tomato because it reduces anther dehiscence, pollen production, and as a consequence fruit set [35]. In several plants, parthenocarpy has been consistently connected to aberrant sexual development. In the apple natural parthenocarpic mutant *Rae Ime*, flowers have no petals or stamens and are unable to complete manual pollination, and these monstrous flowers produce parthenocarpic fruits [36]. In tomato, several mutants and transgenic lines deficient in stamen, anther and ovule development have been described to bear parthenocarpic fruit [37]. The mutant *sha* obtained by mutagenesis with ethyl methane sulfonate has a high tendency for parthenocarpy and abnormalities in anther development [38]. Another tomato mutant, *pat*, is parthenocarpic, sets fruits devoid of seeds and exhibits atypical anther as well as ovule development [35]. The transgenic tomato lines acquired from the processing cultivar ‘UC82’ generate good-quality fruits but seed production was not completely abolished [39]. In this report, we indicate that anthers were bigger than normal and had dehiscence at the top in 35S-RNAi plants while pollen grains were malformed in morphology and defective. Manual pollination with *SlMPK4*-inhibited pollen of WT stigmas could not induce normal ovaries to develop into fruits, indicating male sterility in *SlMPK4*-inhibited plants. As a result, seedless fruits developed in RNAi plants while some had a few seeds. Unexpectedly, LAT52-RNAi plants also developed seedless fruits, as 35S-RNAi plants did. This can be explained from two aspects. According to previous studies, LAT52 is a pollen-specific promoter and is also expressed in seeds and ovaries at a relatively low level [32], which indicated that *SlMPK4* was downregulated in these organs. On the other hand, the exchange of auxin and sugar exists between the anther and embryo, because auxin and sugar homeostasis was affected in RNAi anthers. Auxin and sugar will be changed in LAT52-RNAi embryos, which may affect the young fruit and seed development. The underlying mechanism needs to be further explored. Taking these findings together, we conclude that inhibition of *SlMPK4* results in male sterility and triggers partially parthenocarpic fruit development in tomato. However, we provide new evidence in this report that the parthenocarpy can be triggered by defects in the pollen at late stages in tomato. All these facts confirmed that the defects observed in mature pollen grains of RNAi mutants resulted from the knockdown of *SlMPK4* in tomato. However, ovule development and the female fertility of these transgenic plants were normal. The RNAi plants can produce fruits with fewer seeds or seedless fruits depending on the degree of male fertility. The results provide several lines of evidence that *SlMPK4* is vital for pollen formation and normal development in tomato. In addition, the regulation mechanism needs further experiments.

### Suppression of *SlMPK4* altered the expression of pollen-related genes during flower development

RNA sequencing analysis was used to investigate the downstream genes controlled by *SlMPK4* using *SlMPK4*-RNAi plants driven by the LAT-52 promoter and WT plants. The RNA sequencing analysis found that a total of 1940 genes had dramatically altered expression patterns, with 973 of them upregulated and 996 downregulated in both stages (uninucleate and mature stage), and the highest number of upregulated genes (836) was found at the mature pollen stage (Fig. 9), which was in accordance with the expression level of *SlMPK4* (Fig. 1). This finding provides evidence that *SlMPK4* plays a critical function in late stamen development. Among the DEGs, some encode proteins that are involved in ‘cell differentiation’, ‘cell growth’, ‘cell death’, and ‘carbohydrate and lipid metabolism’, and participate in pollen development. Moreover, the expression patterns of many genes participating in biological processes such as reproduction, embryonic development, post-embryonic development, pollination, pollen–pistil interaction, and flower development were found to be significantly altered. All these findings provide strong evidence for the phenotypes observed in *SlMPK4*-suppressed flowers at the molecular level.

The output of signals in MAPK cascades is determined by the phosphorylation of MAPK substrates such as transcription factors, enzymes, and proteins with other biochemical functions [8]. Several researchers uncovered that the microtubule-associated protein MAP65 is a bridge that links an MAPK to microtubule turnover [40, 41]. Previous studies have shown that *MAP65-1*, *MAP65-2*, and *MAP65-3*/*PLEIADE* function redundantly downstream of MPK4 in *A. thaliana* cytokinesis [16, 42]. Our data showed that *MAP65-1a* was significantly downregulated in *SlMPK4*-inhibited plants, demonstrating that *MPK4* may have conserved roles in cytokinesis in dicots. Our transcriptome analysis of *SlMPK4*-inhibited plants revealed that three ERFs, nine MYBs, and one WRKY had significant changes in their expression pattern, making them possible substrates of *SlMPK4* in tomato pollen development. WRKY/MYB transcription factors have been reported to be involved in N-mediated resistance to tobacco mosaic virus downstream of Ntf6 MAPK [43], and phosphorylation of *WRKY34* by MPK6 has been proved to be essential for pollen development and function in arabidopsis [10]. As a result, we speculated that the WRKY transcription factor that has been identified by transcriptome sequencing may have the greatest possibility of being one of the substrates of *SlMPK4*.

Furthermore, another outstanding finding in this study was that silencing of the *SlMPK4* gene altered the expression of genes controlling starch and sucrose metabolism at transcript levels (Fig. 9 and Table 1). Pollen was symplastically isolated from the rest of the surrounding anther tissues, and hence required import of nutrients via the apoplastic pathway, in which sucrose is first hydrolyzed by cell wall invertases into the monosaccharide sugars glucose and fructose and subsequently transported by sugar transporters into pollen cells [44–46]. While many studies have identified the roles of sugars in pollen maturation and germination stages of pollen grain development, our study proposes similar roles of sugars during the early stages of pollen development, where maintaining optimum assimilation flow and sugar levels are crucial during microsporogenesis as well as microgametogenesis. In our recent study we identified that in the development of pollen in tomato mitotic division was also regulated through auxin signaling and sugar metabolism pathways [47]. Additionally, current studies show that starch and sugar are involved in pollen development, as reported in the articles ‘Deficiency of rice hexokinase HXK5 impairs synthesis and utilization of starch in pollen grains and causes male sterility’ [48] and ‘Comparative analysis of the transcriptome, methylome, and metabolome during pollen abortion of a seedless citrus mutant’ [49]. In line with the above-mentioned concept, UDP-d-glucuronate 4-epimerase 2 (SlUGlcAE2-like) was significantly repressed in *SlMPK4* knockdown anthers during the tetrad and early uninucleate stages. UGlcAE is capable of interconverting UDP-d-galacturonic acid and UDP-d-glucuronic acid. UDP-d-galacturonic acid is the activated precursor utilized in the synthesis of pectin, which forms a major constituent of primary cell walls in plants [50, 51]. Moreover, genes regulating IAA metabolism and signaling are greatly inhibited. To this end, one gene (Solyc02g092820) encoding IAA-amido synthetase and also showing a positive response to exogenous application was significantly downregulated [52] (Table 1). Our results suggest that *SlMPK4* helps pollen growth in a beneficial way, most likely via modulating hormone metabolism and signaling.

### Conclusions

To summarize, we present molecular and cell biological evidence that *SlMPK4* is obligatory for the binucleate-to-mature pollen transition of microgametogenesis in tomato after the first round of pollen mitosis, most likely by modulating the expression of other genes.

## Materials and methods

### Plant material and growth conditions

The ‘Micro-Tom’ tomato cultivar was supplied by the Tomato Genetics Resource Center (University of California, Davis, CA, USA). Selected transgenic and WT tomato plants were grown in growth chambers under cycles of 16 h light (300 μmol photons m^−2^ s^−1^ with a cool white fluorescent lamp) at 25°C and 8 h darkness at 22°C with a relative humidity of 65–75%. Roots, stems, leaves, floral parts at anthesis (sepals, petals, pistils, and stamens), and green mature fruit samples were gathered from fruited tomato plants for expression analysis. Tomato stamens were collected at the following six developmental stages: pollen mother cell (1.8–2.4 mm buds), tetrad (2.5–3.5 mm), early uninucleate (3.6–4.5 mm), late uninucleate (4.6–6.5 mm), binucleate (6.6–7.5 mm) and mature pollen (>7.5 mm flower) [21]. The ovaries and fruits were collected at different developmental stages as follows: unpollinated ovaries at pre-anthesis, corresponding to the binucleate stage (3 days before anthesis); ovaries at anthesis; 3–4, 5–6, 7–8, and 9–10 mm fruits, corresponding to ~6, 8, 10, and 12 days after pollination, respectively; and mature green fruit [53].

### Phylogenetic relationship and sequence alignment

A Blastn and Blastp search was conducted using the NCBI database (http://blast.ncbi.nlm.nih.gov/Blast.cgi) to find heterogeneous homologs of *SlMPK4* (HM367593) [17]. A multiple sequence alignment of plant *MPK4* genes was generated using Clustal X v1.81 with default settings [54]. The phylogenetic tree was generated by iTOL (https://itol.embl.de/) making use of the neighbor-joining method comprising 1000 bootstrap replications [55, 56].

### Prokaryotic expression and MAPK activity assay

The open reading frame (ORF) fragment of *SlMPK4* was cloned into the pET32a (+) vector using primers digested with BaxmH I and Hind III (primer pairs are shown in Supplementary Data Table S1). Small His-Tag, Trx-Tag, and S-Tag sequences were harbored in the fusion protein. The recombinant vector was then introduced into *Escherichia coli* strain DE3. The expression of *SlMPK4* fusion protein was induced at 30°C after adding 0.5 mmol l^−1^ of isopropyl-β-d-thiogalactoside in *E. coli* cells, and then cultured for further 4 hours. The His-Bind Kit (NovaGen, Madison, WI, USA) was used to purify the fusion protein following the manufacturer’s instructions, and the protein was identified using an anti-His antibody on a western blot (Huaan, Hangzhou, China). Under the same conditions, the empty pET32a (+) vector was also induced. MAPK activation was detected by immune blot analysis using anti-pTEpY (antiphospho-p44/42-ERK; Cell Signaling Technology, http://www.cellsignal.com/) as previously described [25, 26].

### Protein localization of SlMPK4

Transient expression was achieved using tobacco epidermal cells to detect the localized protein of the target gene [55], followed by the ORF segment of SlMPK4 (primers are shown in Supplementary Data Table S1) fused into the pVBG2307 vector controlled by the CaMV 35S promoter then transformed into GV3101 (*Agrobacterium tumefaciens*) strain. The empty vector of pVBG2307:GFP was used as a control check. Four-week-old tobacco plantlets expressing the red nuclear marker RFP-H2B were agroinfiltrated and then placed in a growth chamber for 2–3 days [57]. Localized protein expression was scanned with an Olympus BX63 automated fluorescence microscope (Olympus, Tokyo, Japan).

### Quantitative RT–PCR

Isolation of total RNA and synthesis of first-strand cDNA were conducted as explained previously [55]. Real-time RT–PCR analysis was used to analyze the expression of *SlMPK4*. Supplementary Data Table S1 lists all the primers used in the expression analyses. Each sample was tested with three biological and three technical replicates. The *SlUbi3* gene (accession number X58253) was amplified synchronously as an endogenous control for the calibration of relative expression. The 2^-△△^CT method [58] was employed in calculating the relative expression levels of genes in different treatments.

### Overexpression/RNAi constructs and transformation

Two kinds of *SlMPK4* RNAi constructs were designed in which the transcription of a hairpin-shaped *SlMPK4* mRNA fragment was driven by a constitutive CaMV 35S promoter or the pollen-specific promoter LAT52 [29, 30, 34]. A 273-bp sense fragment and another 120-bp antisense fragment were amplified using primer pairs (Supplementary Data Table S1). They were cloned into p35S::pCAMBIA1301 and pLAT52::pCAMBIA1301 using specific restriction endonucleases in sense or antisense orientation. Finally, two different RNAi constructs, namely, p35S::*SlMPK4*-RNAi and pLAT52::*SlMPK4*-RNAi, were confirmed by PCR and sequencing. For overexpression of *SlMPK4*, the target fragment of *SlMPK4* was amplified and inserted into the pCAMBIA1301 vector driven by the CaMV 35S promoter. All the fusion vectors, including p35S::*SlMPK4*-RNAi, pLAT52::*SlMPK4*-RNAi, and p35S::*SlMPK4*, as well as an empty control vector, p35S::pCAMBIA1301, were transformed into *A. tumefaciens* GV3101. Tomato transformation using cv. ‘Micro-Tom’ was performed by leaf-disk methods, as described in a previous study [59]. At least 12 independent lines harboring a single T-DNA insertion from each construct were generated, and at least three independent lines of their *T*_2_ plants were used for comprehensive analysis.

### PCR analysis and histochemical β-glucuronidase assay

The cetyl trimethyl ammonium bromide extraction technique was used to isolate genomic DNA from fresh young tomato leaves [60]. PCR was carried out to detect an ~600-bp fragment of the *HPT* gene in the pCamiba1301 vector, which has the *GUS* reporter gene, to confirm the presence of a transgenic cassette. Leaves, flowers, and roots of flowering transgenic and control tomato plants were harvested and incubated in GUS solution at 37°C overnight [61]. A Leica MZ16 FA fluorescence stereomicroscope was used to take photomicrographs.

### Pollen germination and phenotypic analysis

To analyze pollen viability, pollen grains were obtained from the anthers and stained using the Alexander procedure [62]. The pollen nuclei were stained with DAPI solution and observed by fluorescence microscopy [63]. For pollen germination *in vitro*, pollen grains were collected and incubated in a germination medium containing 15% sucrose (w/v), 0.4 mmol l^−1^ HBO_3_, 0.4 mmol l^−l^ Ca(NO_3_)_2_, and 0.1% agar (w/v) (pH 5.8) at 20–25°C and 100% relative humidity in the dark for at least 4 hours and the germination frequencies were estimated. For all experiments, three independent transgenic lines were examined. For each plant, pollen from >10 flowers was tested, and at least 300 pollen grains were counted. Photographs were taken with a Nikon Eclipse 90i microscope.

### Semithin section and electron microscopy

For SEM, individual pollen grains collected from *SlMPK4* transgenic plants and controls were spread on SEM (Philips XL-40) stubs and coated with gold–palladium in an Eiko Model IB5 ion coater for 4–5 minutes, and then vacuum desiccation scanning electron micrographs were acquired using a Hitachi Model TM-1000 scanning electron microscope.

For semi-ultrathin section microscopy and TEM, the anthers at various developmental stages were prefixed with 2.5% glutaraldehyde overnight and rinsed in 0.1 M phosphate buffer. They were then transferred into 1% osmic acid for 1 hour and then washed again in phosphate buffer as explained in our recently published paper [21] and then dehydrated through a graded series of ethanol concentrations. Samples were embedded in Spurr resin. Anthers were cut transversely into semi-ultrathin and ultrathin sections. The semi-ultrathin sections (2 μm) were stained with dimethyl blue and observed under a Nikon Eclipse 90i microscope. Ultrathin sections (50 nm) were double-stained with 2% (w/v) uranyl acetate followed by alkaline lead citrate. Digital images were then observed and recorded with a Hitachi ModelH-7650 transmission electron microscope.

### Transcriptome sequencing

Anthers of tomato plants at two different developmental stages were sampled from RNAi transgenic lines under the LAT52 promoter and from the control, then immediately placed in liquid nitrogen. Total RNA extraction was carried out using TRIzol reagent following the manufacturer’s procedures (Invitrogen). Strand-specific RNA libraries were constructed using 10 μg of total RNA according to a previously described protocol [64]. RNA sequencing was performed on three replicates of each sample using the Illumina HiSeq2000 system.

### RNA sequencing analysis

First, the Illumina raw RNA sequencing reads were analyzed to eliminate low-quality and adaptor contaminant sequences. Then, the reads were aligned to ribosomal RNA (rRNA) and transfer RNA (tRNA) sequences using Bowtie [65] to remove potential ribosomal and tRNA contaminating reads, allowing up to three mismatches. The filtered reads were then aligned to the tomato genome with TopHat [66], allowing one segment mismatch. Raw counts of mapped reads for each gene model of tomato were derived after alignments and then normalized to FPKM (fragments per kilobase of transcript per million mapped reads). For the expression analyses of selected genes from transgenic and control flowers, the raw counts of RNA sequencing expression data were used to find genes that were differentially expressed with the DESeq package [67]. The variable and stable transformed expression data were then supplied to the LIMMA package [68]. *F*-tests were then executed and raw *P*-values were adjusted using the Benjamini–Hochberg procedure for multiple testing [69]. Differentially expressed genes were fed to the Plant MetGenMAP system database to identify enriched GO terms. Metabolic pathway analysis was conducted using Blastx/Blastp 2.2.24+ in KEGG (http://www.genome.jp/kegg/genes.html) [70, 71].

## Acknowledgements

We highly appreciate the financial support of the National Key Research and Development Program of China (2019YFD1000302), the National Natural Science Foundation of China (31772316), the Key Research and Development Program of Zhejiang Province (2021C 02052), and the Starry Night Science Fund of Zhejiang University Shanghai Institute for Advanced Study (SN-ZJU-SIAS-0011).

## Author contributions

G.L. conceived and designed the research. J.W and M.L. conducted the experiments. S.Z., Y.L., X.Y., S.M., and M.A. analyzed the data. M.A. and G.L. wrote and critically revised the manuscript. G.L. contributed reagents and funded the project. All the authors read and approved the manuscript.

## Data availability

All datasets generated for this study are included in the article and supplementary materials.

## Conflict of interest

The authors declare that the research was conducted in the absence of any commercial or financial relationships that could be construed as a potential conflict of interest.

## Supplementary data

Supplementary data is available at *Horticulture Research* online.

## Supplementary Material

Web_Material_uhac048
