# Supplementary material for: Mitogen-activated protein kinase 4 is obligatory for late pollen and early fruit development in tomato
Source: Hortic Res. 2022 Mar 14;9:uhac048. doi: 10.1093/hr/uhac048 (PMC9113226; doi:10.1093/hr/uhac048)
Supplement: Web_Material_uhac048 [file web_material_uhac048.zip › Supplementary Tables.docx]

**Mitogen-activated protein kinase 4 is obligatory for late pollen and early fruit development in tomato**

Jie Wang^1,2†^, Mengzhuo Li^1†^, Shibin Zhuo^1^, Yue Liu^1^, Xiaolin Yu^1^, Sidra Mukhtar^3^, Muhammad Ali*^1^, Gang Lu*^1,4^

^1^Department of Horticulture, Zhejiang University, Hangzhou 310058, China

^2^Ningbo Academy of Agricultural Sciences, Ningbo 315000, Zhejiang, China

^3^Department of Horticulture, The University of Agriculture Peshawar, Pakistan

^4^Key Laboratory of Horticultural Plant Growth, Development and Quality Improvement, Ministry of Agricultural, Zhejiang University, Hangzhou 310058, China

^†^These authors contributed equally to this work

*Corresponding Authors: [maur202@zju.edu.cn](mailto:maur202@zju.edu.cn) and [glu@zju.edu.cn](mailto:glu@zju.edu.cn)

**Table S1.** Primer pairs

|  |  |  |
| --- | --- | --- |
| qRT-PCR | F | TTCCGAGCATTTCCCTGAT |
|  | R | TGGTTCCTCGTTTATCTCAT |
| In-situ | F | CGCGGATCCGGATTCCTAAGGAGTGACA |
|  | R | TGCTCTAGATAAGAGCCTCGTTCCATAT |
| Western blot | F | GGATCCATGGATGCTGAAAACATTGAAAATTCAGTGG |
|  | R | AAGCTTTCACTTGGTTGTATCGGGATCAAACTTA |
| Subcellular localization | F | CGCGGATCCATGGATGCTGAAAACATTGAA |
|  | R | TGCTCTAGAGCACTTGGTTGTATCGGGAT |
| RNAi-sense probe | F | CGCGGATCCAATTCCTACTCGGGATGGT |
|  | R | TGCTCTAGACCAAAACTTCCTCAGACCAA |
| RNAi-Antisense probe | F | CCCAAGCTTAATTCCTACTCGGGATGGT |
|  | R | TGCTCTAGACAGACGATTCCGTAAGCTC |
| OE | F | AAGCTTATGGATGCTGAAAACATTGAA |
|  | R | GGATCCGCACTTGGTTGTATCGGGAT |
