# Supplementary material for: Mitogen-activated protein kinase 4 is obligatory for late pollen and early fruit development in tomato
Source: Hortic Res. 2022 Mar 14;9:uhac048. doi: 10.1093/hr/uhac048 (PMC9113226; doi:10.1093/hr/uhac048)
Supplement: Web_Material_uhac048 [file web_material_uhac048.zip › Supplementary Figures .docx]

**Mitogen-activated protein kinase 4 is obligatory for late pollen and early fruit development in tomato**

Jie Wang^1,2†^, Mengzhuo Li^1†^, Shibin Zhuo^1^, Yue Liu^1^, Xiaolin Yu^1^, Sidra Mukhtar^3^, Muhammad Ali*^1^, Gang Lu*^1,4^

^1^Department of Horticulture, Zhejiang University, Hangzhou 310058, China

^2^Ningbo Academy of Agricultural Sciences, Ningbo 315000, Zhejiang, China

^3^Department of Horticulture, The University of Agriculture Peshawar, Pakistan

^4^Key Laboratory of Horticultural Plant Growth, Development and Quality Improvement, Ministry of Agricultural, Zhejiang University, Hangzhou 310058, China

^†^These authors contributed equally to this work

*Corresponding Authors: [maur202@zju.edu.cn](mailto:maur202@zju.edu.cn) and [glu@zju.edu.cn](mailto:glu@zju.edu.cn)


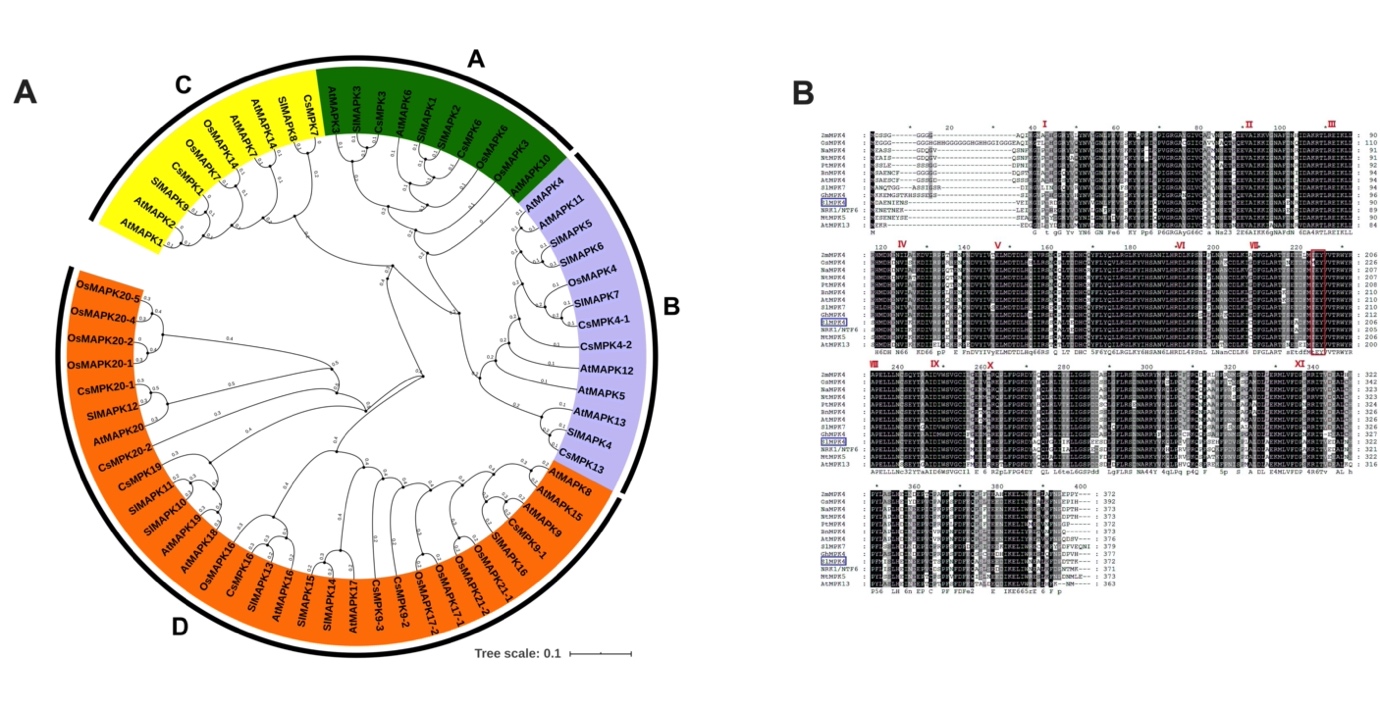


**Figure S1.** Web based analysis of *MAPK4* gene. **(A)** Molecular phylogenetic tree of the MAPK family genes in tomato (*Solanum lycopersicum*) and *Arabidopsis thaliana* as well as the homologs of *SlMPK4* in other plant species. The unrooted tree was generated using online tool iTOL (<https://itol.embl.de/>) with the neighbor-joining method. Bootstrap values (above 50%) from 1000 replicates are indicated at each branch. **(B)** Alignment of predicted amino acids from *SlMPK4* homolog. The highlighted residues show the conserved signature motif obtained with the ClustalX program. Roman numerals (I–XI) indicate conserved domains that are found in plant serine/threonine protein kinasese. The “TXY” motif marked in the red square shows residues corresponding to the sites of regulatory phosphorylation in plant MAP kinases.

**
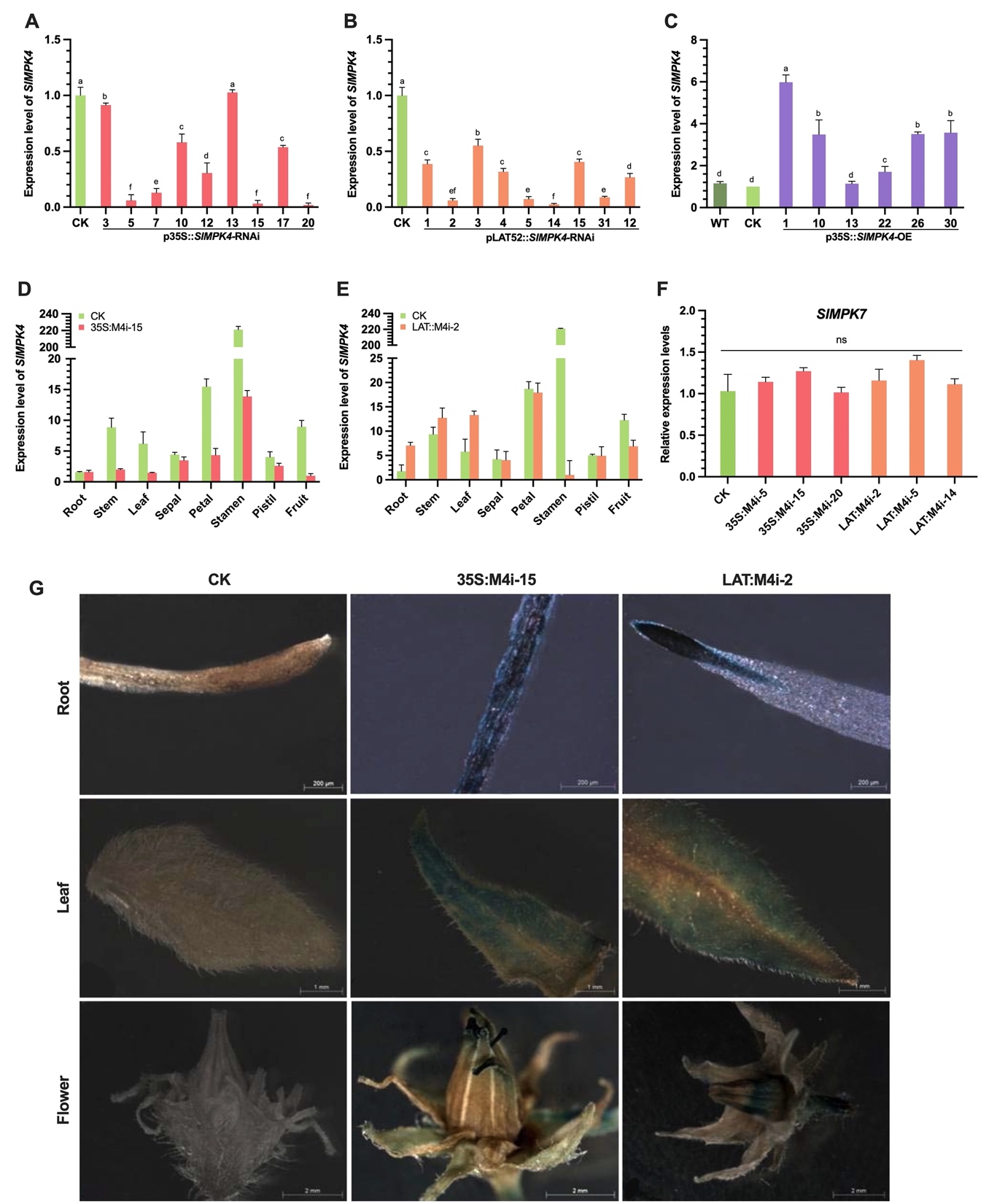
**

**Figure S2.** Expression pattern of SIMAPK4 in tomato plant **(A)** Silencing efficiency of *SIMAPK4* in p35S:MAPK4-RNAi lines (35S:M4i-3, 35S:M4i-5, 35S:M4i-7, 35S:M4i-10, 35S:M4i-12, 35S:M4i-13, 35S:M4i-15, 35S:M4i-17, 35S:M4i-20) **(B)** Silencing efficiency of *SIMAPK4* in pLAT52:RNAi lines (LAT:M4i-1, LAT:M4i-2, LAT:M4i-3, LAT:M4i-4, LAT:M4i-5, LAT:M4i-14, LAT:M4i-15, LAT:M4i-31, LAT:M4i-12) **(C)** The relative expression level of *SIMAPK4* under normal condition in WT and *SIMAPK4*-overexpressed lines (OE:M4-1, OE:M4-10, OE:M4-13, OE:M4-22, OE:M4-26, OE:M4-30) **(D)** Transcript levels of *SIMAPK4* in different tissues of 35S:M4i-15 and CK plants, all are significant relative to their CK **(E)** Transcript levels of *SIMAPK4* in different tissues of LAT:M4i-2 and CK plants, all are significant relative to their CK **(F)** Relative expression levels of *SlMPK7* in CK, 35S:*SlMPK4*-RNAi, LAT52:*SLMPK4*-RNAi, p35S::*SLMPK4* lines **(G)** GUS histochemical assay of CK, 35S:M4i-15 and LAT:M4i-2 tomato plants in root, leaf and flower. Different tissues were dyed with GUS solution at 37 °C overnight, and then photos were captured. Blue color showed GUS in root, leaf and flower of 35S:M4i-15 and LAT:M4i-2. Note: Figure A, B, C and F used stamens of mature stage flowers for the detection of silencing efficiency. ﻿ Plotted values are mean (3 biological replicates) ± standard deviation and separated using Duncan’s Multiple Range (DMR) test (P<0.05); means with different lower-case (a–f) letters represent significant differences.

**
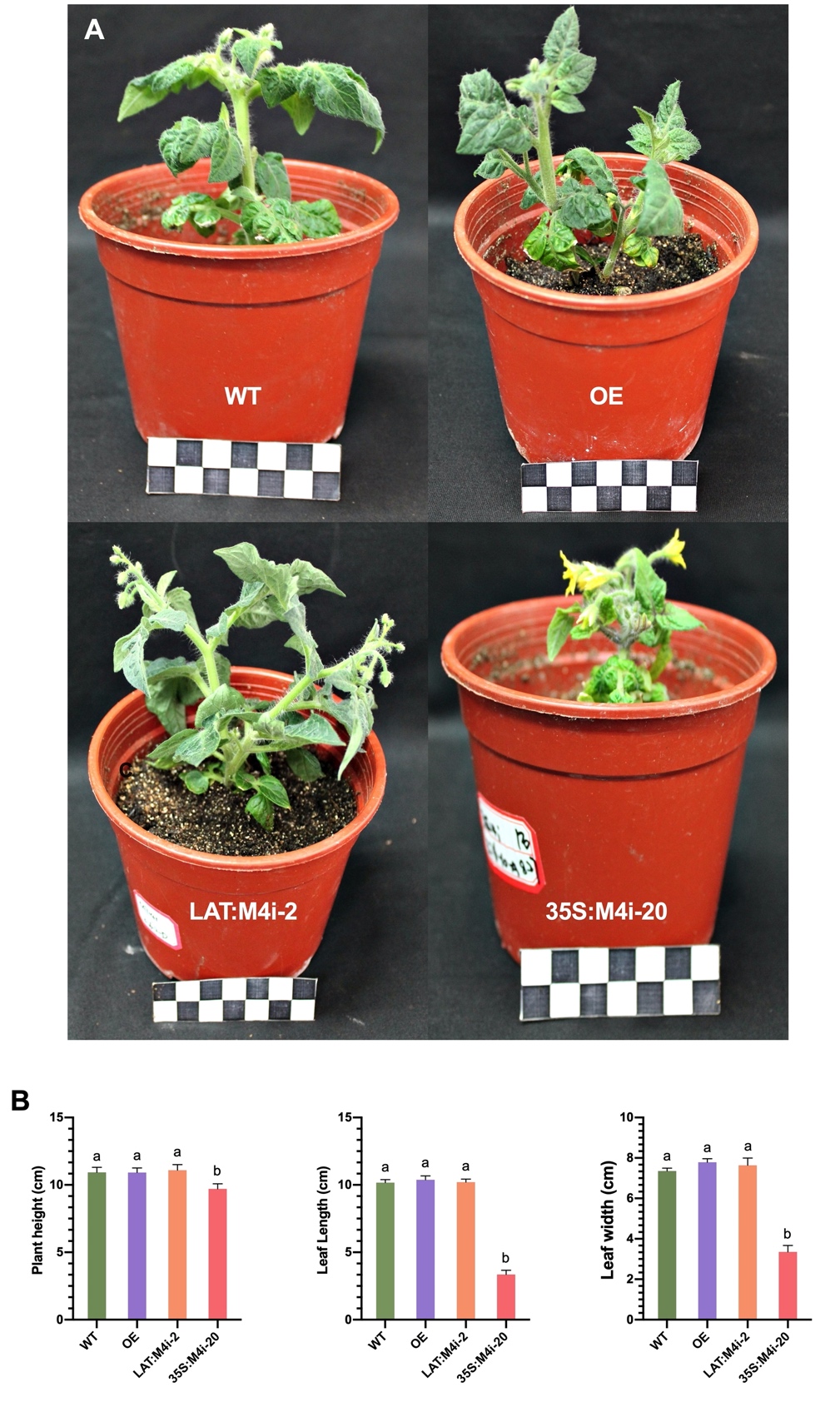
**

**Figure S3.** Vegetative growth of *SlMPK4*-overexpressed, *SlMPK4*-silenced and control plants (A) Phenotypes (B) Quantitative data of plant height, leaf length and leaf width. The samples were collected in three biological replicates. Plotted values are mean ± standard deviation and separated using Duncan’s Multiple Range (DMR) test (P<0.05); means with different lower-case (a–b) letters represent significant differences.

**
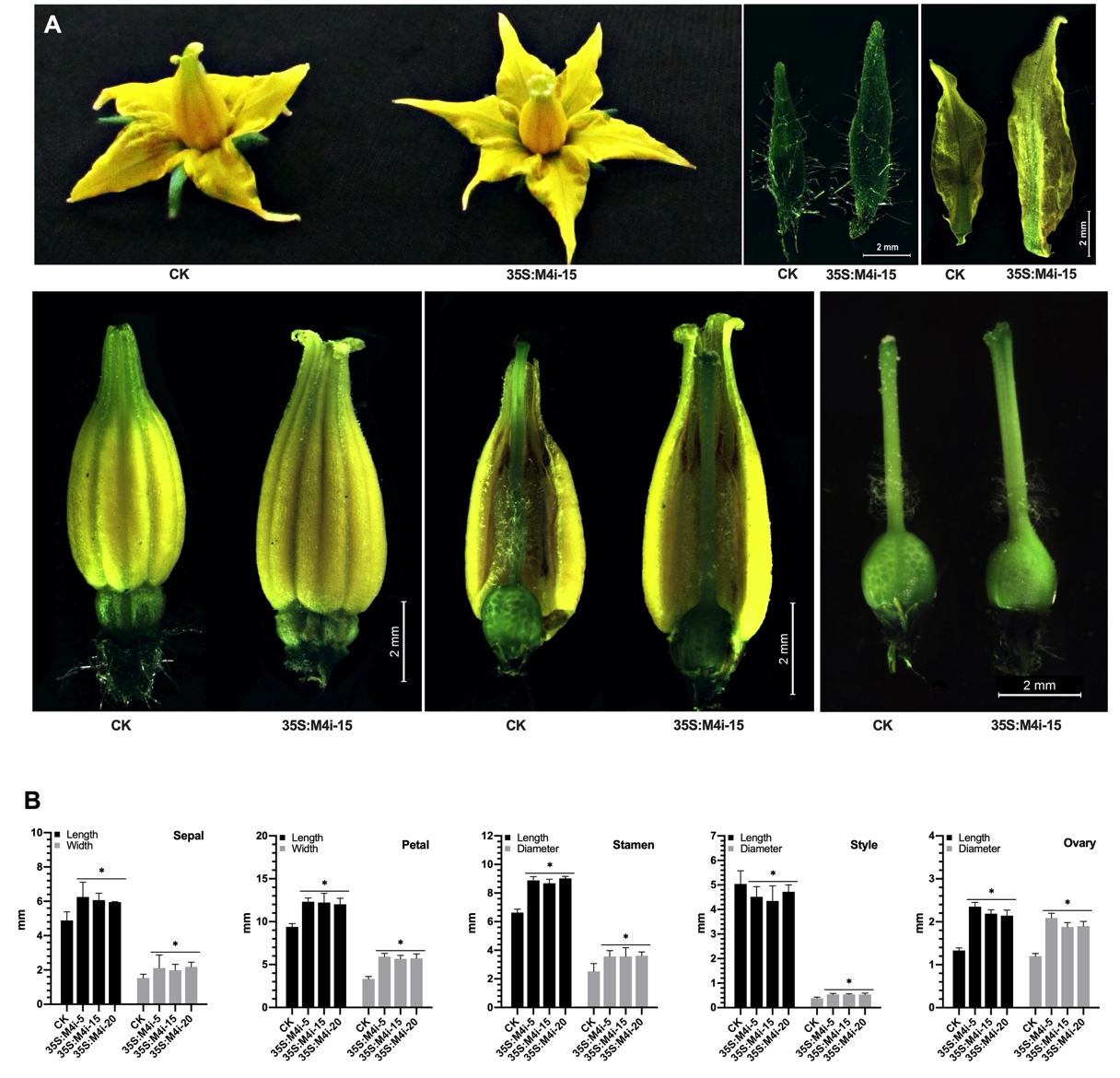
**

**Figure S4.** Characterization of opened flower in transgenic tomato. (A) Morphological observation of the entire opened flower; sepal morphology of opened flower; petal morphology of opened flower; stamen morphology of opened flower; stamen and pistil morphology of opened flower; stigma and ovary morphology of opened flower (B) Graphical representation of the data. Plotted values are mean (3 biological replicates) ± standard deviation and separated using Duncan’s Multiple Range (DMR) test (P<0.05); means with asterisk (*) represent significant differences.

**
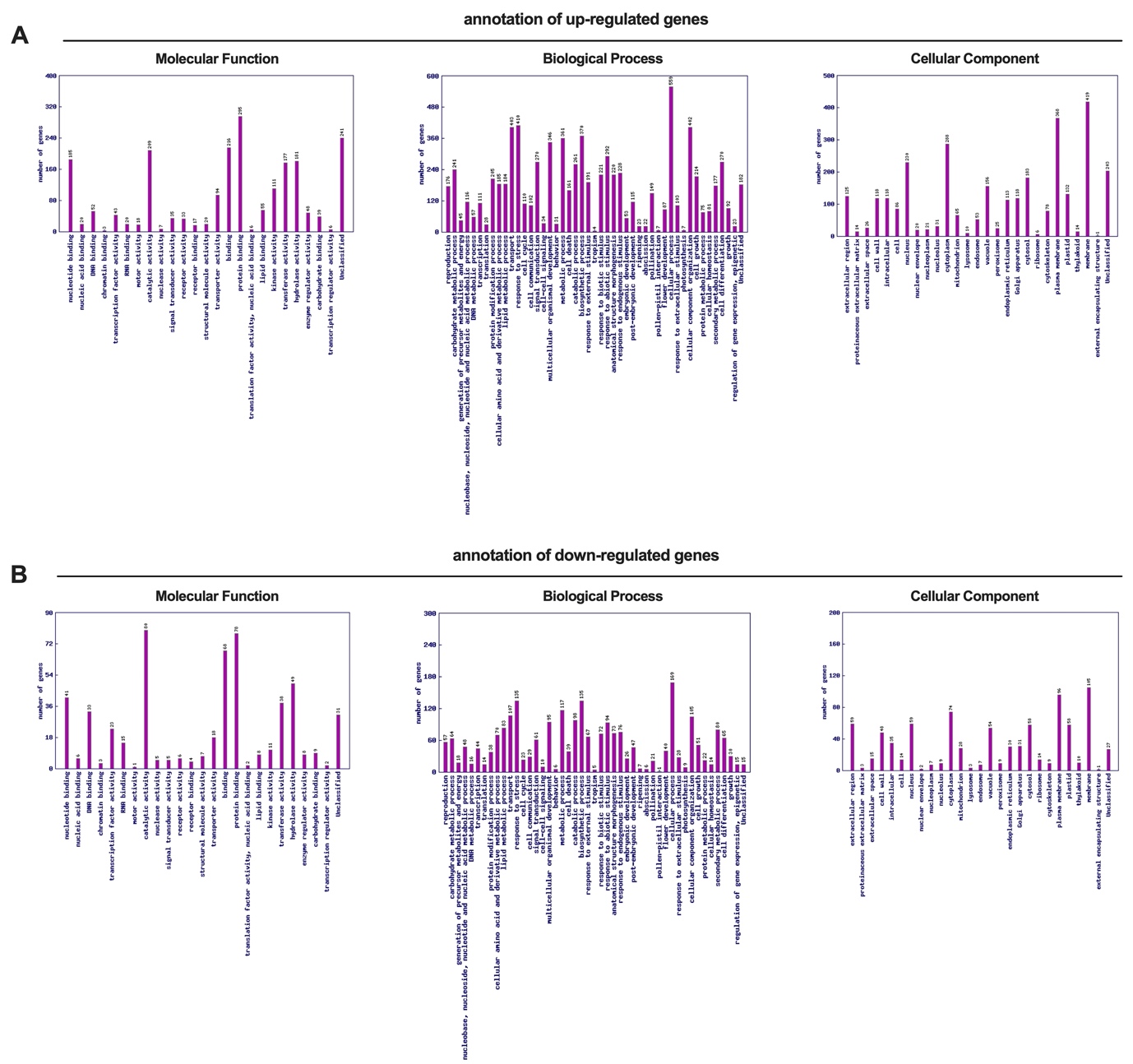
**

**Figure S5.** Gene ontology analysis of up- and down-regulated genes in three different categories. (A) Annotation of up-regulated genes (B) Annotation of down-regulated genes.

**
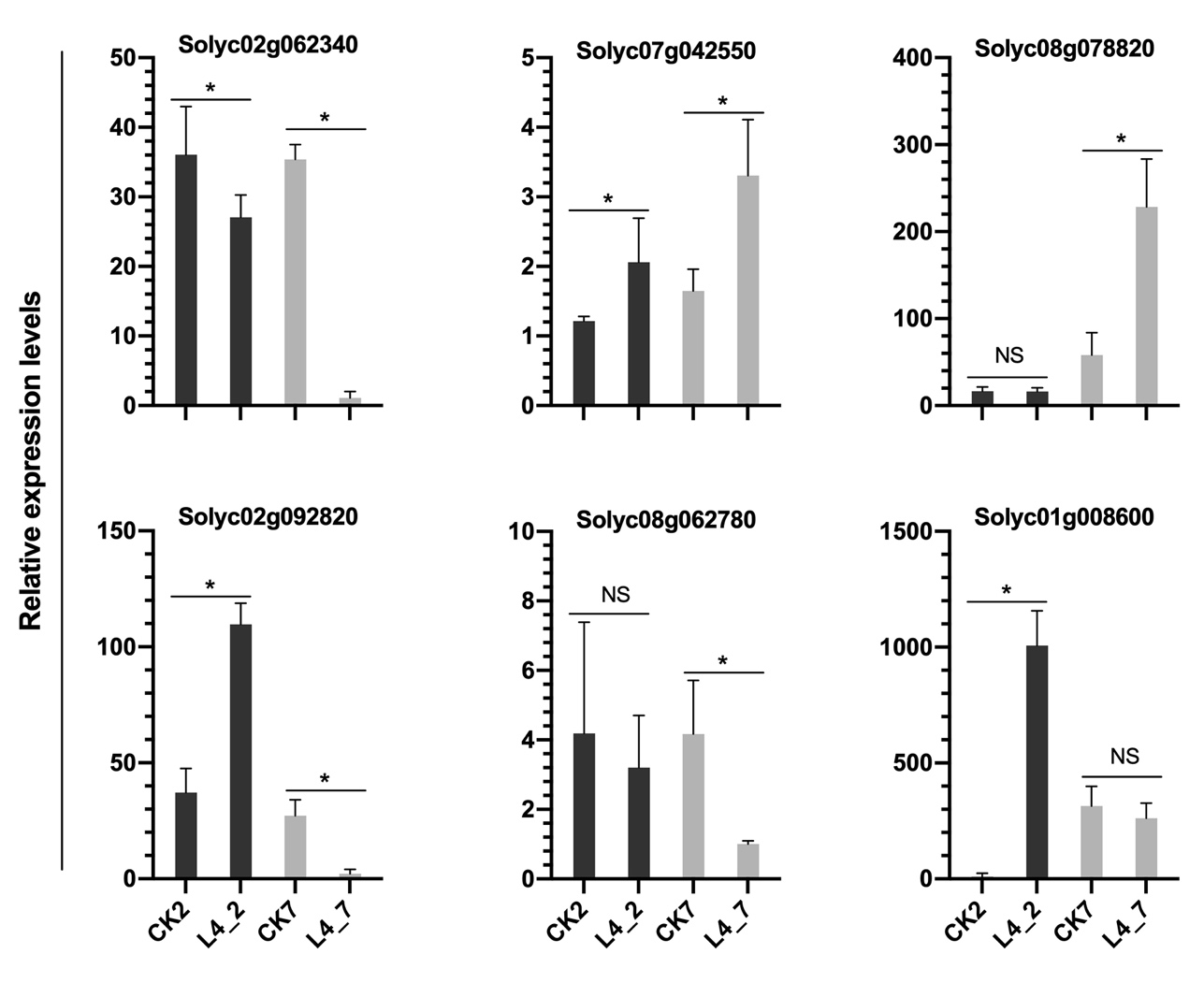
**

**Figure S6.** qRT-PCR analysis of downstream genes. Mean values and standard deviation (+SD) are plotted as analyzed by Duncan’s multiple range (DMR) test (P < 0.05); asterisk (*) indicates signiﬁcant difference.
